# Mechanisms involved in VPAC receptors activation and regulation: lessons from pharmacological and mutagenesis studies

**DOI:** 10.3389/fendo.2012.00129

**Published:** 2012-10-30

**Authors:** Ingrid Langer

**Affiliations:** Institut de Recherche Interdisciplinaire en Biologie Humaine et Moléculaire, Faculté de Médecine, Université Libre de BruxellesBrussels, Belgium

**Keywords:** VIP, VPAC_1_, VPAC_2_, mutagenesis, activation, signaling, regulation, oligomerization

## Abstract

Vasoactive intestinal peptide (VIP) plays diverse and important role in human physiology and physiopathology and their receptors constitute potential targets for the treatment of several diseases such as neurodegenerative disorder, asthma, diabetes, and inflammatory diseases. This article reviews the current knowledge regarding the two VIP receptors, VPAC_1_ and VPAC_2_, with respect to mechanisms involved in receptor activation, G protein coupling, signaling, regulation, and oligomerization.

## VASOACTIVE INTESTINAL PEPTIDE

Vasoactive intestinal peptide (VIP) is a 28 amino acids peptide isolated from porcine intestine (Said and Mutt, 1970) and that belongs to a family of structurally related peptide hormones that includes secretin, pituitary adenylate cyclase-activating polypeptide (PACAP), glucagon, glucagon-like peptides (GLP), gastric inhibitory peptide (GIP), and growth hormone-releasing hormone (GHRH). It has initially been shown that VIP has a diverse range of effects such as vasodilation, relaxation of smooth muscle in intestinal and pulmonary tissues, and stimulation of electrolyte secretion in the gut (Dickson and Finlayson, 2009; Harmar et al., 2012). As a consequence, VIP was first classified as a gut hormone. Later studies demonstrated that VIP has a more expanded distribution including peripheral (PNS) and central (CNS) nervous system (Larsson et al., 1976a,b) as well as cells and tissues of the immune system (Delgado et al., 2004), suggesting that VIP may also behave as a neuroendocrine hormone, a neurotransmitter and a cytokine-like peptide. Many studies supported this hypothesis and it is now well accepted that VIP plays important role in the CNS such as control of circadian rhythms, anxiety, response to stress, schizophrenia, learning, and memory (King et al., 2003; Dickson and Finlayson, 2009; Moody et al., 2011; Harmar et al., 2012). In the periphery, it is involved in the control of insulin secretion from the pancreas and the release of catecholamines from the adrenal medulla (Dickson and Finlayson, 2009; Moody et al., 2011; Harmar et al., 2012). VIP also acts as a co-transmitter of non-adrenergic non-cholinergic relaxation of vascular and non-vascular smooth muscles (Said and Rattan, 2004). Finally, in the immune system VIP has potent effects on T cell differentiation and migration and modulates cytokine production by T helper cells (Delgado et al., 2004).

## VPAC RECEPTORS

The biological effects of VIP are mediated by two receptors named VPAC_1_ and VPAC_2_ that belong to the family B of G protein-coupled receptors (GPCRs) which also includes PAC_1_-, secretin-, glucagon-, GLP 1 and 2-, calcitonin-, GIP-, corticotropin-releasing factor (CRF) 1 and 2-, and parathyroid hormone receptors. Besides VIP, VPAC_1_ and VPAC_2_ receptors also bind with the same high affinity PACAP. The VPAC_1_ receptor was initially cloned from rat lung (Sreedharan et al., 1993) and the VPAC_2_ receptor from rat pituitary (Lutz et al., 1993). Like VIP, VPAC receptors are widely distributed throughout the body. In the CNS, VPAC_1_ receptors are abundantly localized in piriform cortex, cerebral cortex, suprachiasmatic nucleus, hippocampus, and pineal gland (Usdin et al., 1994) while VPAC_2_ receptors are mainly found in cerebral cortex, suprachiasmatic nucleus, thalamus, hypothalamus, and amygdala (Sheward et al., 1995). Although both receptors may be co-expressed in the same areas of the CNS, studies suggest that the two receptor subtypes have complementary distribution. In peripheral tissues, VPAC_1_ receptors have been found in breast, kidney, liver, lung, prostate, spleen, and mucosa of the gastrointestinal tract (Reubi, 2000). Similarly, VPAC_2_ receptors have a large distribution and have been localized in adrenal medulla, blood vessels, lung, pancreatic acinar cells, smooth muscle, and thyroid follicular cells (Harmar et al., 2004). In the immune system, VPAC_1_ receptors are constitutively expressed in T cells, monocytes, and macrophages while expression of VPAC_2_ receptors is induced upon activation of T cells and macrophages (Delgado et al., 2004).

Like all members of the GPCR-B family, VPAC receptors are heptahelical membrane proteins and are characterized by the presence of a large N-terminal extracellular domain. The recent solving of the NMR or X-ray structure of the N-terminus of several family B receptors [CRF, PTH, PAC_1_, GIP, GLP-1, CLR/RAMP1, and VPAC_2_(PDB ID: 2X57)] clarified their role in ligand binding. The structure comprises a crucial sushi domain characterized by two antiparallel β sheets and stabilized by three disulfide bonds and a salt bridge sandwiched between aromatic rings of two tryptophan residues (Harmar et al., 2012). The data support the two-site model for peptide binding to family B GPCR, in which the N-terminal domain of the receptor is the principal binding site for the central and the C-terminal regions of the natural ligand and ensures correct ligand positioning, whereas binding of residues 1–6 of the ligand to the extracellular loops and transmembrane helices drives the receptor activation (Hoare, 2005). More recently, it has also been proposed that a helix N-capping motif, identified in the N-terminus of all GPCR-B family ligands and stabilizing their helical conformation, was probably formed upon receptor binding and could also constitute a key element in receptor activation (Neumann et al., 2008). Although the experimental structure of the VPAC_1_ has not been solved, a three-dimensional model obtained by homology modeling associated with photoaffinity experiments supports that VPAC_1_ shares the same features (Couvineau and Laburthe, 2012).

## SIGNALING PATHWAYS ACTIVATED BY VPAC RECEPTORS

**FIGURE 1:**
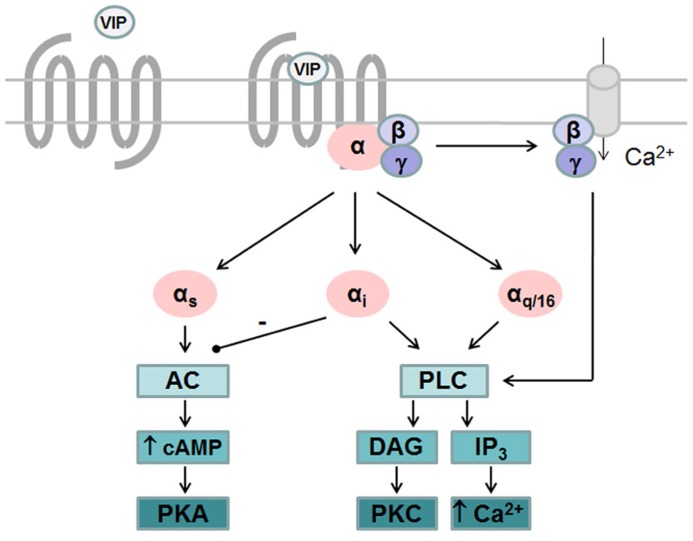
**G protein-dependent signaling pathways activated by VPAC receptors**. VPAC receptors are preferentially coupled to Gαs leading to activation of adenylate cyclase (AC) and subsequent cAMP production and activation of protein kinase A (PKA). VPAC receptors are able to activate phospholipase C (PLC) pathway after coupling to Gαi, Gαq, or Gα_16_ and stimulate calcium levels and protein kinase C (PKC). Gαi-dependent activation of PLC by VPAC_2_ relies on the availability of free Gβγ and on Ca^2^^+^ entry through receptor-operated Ca^2^^+^ channels while Gαi-dependent activation of PLC by VPAC_1_ is not affected by chelating of extracellular calcium.

Like all GPCRs, upon agonist binding VPAC receptors undergo physical/conformational changes that allow interaction of cytoplasmic domains with heterotrimeric G proteins and promote exchange of GDP for GTP on the Gα subunit. This initiates the dissociation of GTP-bound Gα subunit from Gβγ dimer and activation of downstream effector pathways. Signals are terminated following hydrolysis of Gα-bound GTP and reformation of inactive trimeric complex (Pierce et al., 2002). VPAC receptors are preferentially coupled to Gαs leading to activation of adenylate cyclase and subsequent cAMP production. Accumulation of intracellular cAMP also leads to activation of protein kinase A (PKA) that may activate the ERK signaling pathway to promote proliferation (Pechon-Vallee et al., 2000) and neuroendocrine cell differentiation (Gutierrez-Canas et al., 2005) as seen in pituitary cells and prostate cancer cell line, respectively. VIP-induced PKA activation is also responsible for most of the anti-inflammatory activity of VIP by regulating several signaling pathways and transcription factors thus increasing anti-inflammatory cytokines and reducing pro-inflammatory cytokines production (Gonzalez-Rey et al., 2007). Several studies also reported that VPAC receptors were able to activate phospholipase C (PLC) pathway and stimulate calcium levels, either in cells endogenously expressing VPAC receptors or in transfected cell lines. However, the precise mechanisms which contribute to VIP-induced [Ca^2^^+^]_i_ increase remain unclear due to divergent results. Indeed, in transfected cell lines some studies observed that activation of PLC was partly sensitive to pertussis toxin (PTx; MacKenzie et al., 1996; Langer et al., 2002), thus involving both Gαi and Gαq coupling, while others did not observe coupling to Gαi (Sreedharan et al., 1994). Moreover, PTx sensitive mechanisms leading to PLC activation seems different for VPAC_1_ and VPAC_2_ receptors. Cross-linking studies demonstrated that physical interaction between VPAC_1_ and Gαi and VPAC_1_-induced [Ca^2^^+^]_ i_ increase are not affected by chelating of extracellular calcium (Langer et al., 2002), while PTx sensitive activation of PLC by VPAC_2_ relies on the availability of free Gβγ and on Ca^2^^+^ entry through receptor-operated Ca^2^^+^ channels (MacKenzie et al., 2001). Finally, it was also observed that both VPAC_1_ and VPAC_2_ are able to couple to Gα16, a G protein of the Gαq family, that enables the coupling of a wide variety of receptors to PLC and whose expression is restricted to hematopoietic cells (with the exception of the mature B cells; Langer et al., 2001). These findings indicate that the specific G protein/second messenger complement of the cell line/type being examined could alter the transduction pathways/pharmacology observed for VPAC receptors (**Figure 1**).

Additional coupling events that are not G protein-mediated may also elicit auxiliary signals. Both VPAC_1_ and VPAC_2_ are able to activate phospholipase D (PLD). PLD responses induced by VPAC_2_ are not affected by PLC inhibitors, PTx or PKA inhibitors but are sensitive to brefeldin A an inhibitor of ADP-ribosylation factor (ARF) known to act as a direct activator of PLD (McCulloch et al., 2000). In pancreatic β cells, VIP induced sustained stimulation of insulin secretion is mediated by phosphatidylinositol 3 kinase activation by VPAC_2_ receptors (Dickinson et al., 1999). In the rat pineal gland, VIP stimulates cGMP formation through activation of nitric oxide synthase, NO production and activation of cytosolic guanylate cyclase (Spessert, 1993).

## MOLECULAR MECHANISMS INVOLVED IN VPAC RECEPTORS ACTIVATION

The recent solving of the X-ray crystal structures of several GPCR-A family members in complex with agonist, antagonist, and G proteins provides clues to the transmembrane helix (TM) rearrangements that result from agonist binding and subsequent receptor activation. These include the disruption of an ionic interaction involving the E/DRY motif located at the cytoplasmic face of TM3 and maintaining the receptor preferentially in a ground inactive conformation in absence of agonist (ionic lock), a “rotamer toggle switch” (modulation of the helix conformation around a proline kink) in TM6 causing key sequences to be exposed to cytoplasmic binding partners and a conformational change of Y residue of the NPXXY motif located in TM7 stabilizing the active conformation (Rosenbaum et al., 2009, 2011; Rasmussen et al., 2011). Surprisingly, the predicted interaction between E/DRY motif located in TM3 and a glutamate residue located in TM6 (“ionic lock”) was solely observed in rhodopsin inactive state crystals but not in other GPCR crystals available to date. Instead, as seen for the β2-adrenergic receptor (β2-AR), Arg of the E/DRY motif interacts primarily with the adjacent Asp residue in crystals of β2-AR stabilized with the inverse agonist carazolol (inactive conformation; Rasmussen et al., 2007; Rosenbaum et al., 2007) while in crystals of β2-AR/Gs protein complex (active conformation) this interaction is broken and Arg of the E/DRY motif packs against a Tyr residue of Gs (Rasmussen et al., 2011). In absence of X-ray crystal structure of the VPAC receptors, only model structures of VPAC_1_ have been reported which used as template the structures of the N-terminal domain of the CRF 2β receptor (Ceraudo et al., 2012) or structures of family A GPCRs for the transmembrane domains (Conner et al., 2005; Chugunov et al., 2010). However, the low sequence identity between the sequence of the VPAC receptors and the templates used for homology modeling prevents direct transposition of molecular switches that drive GPCR-A members activation.

**FIGURE 2:**
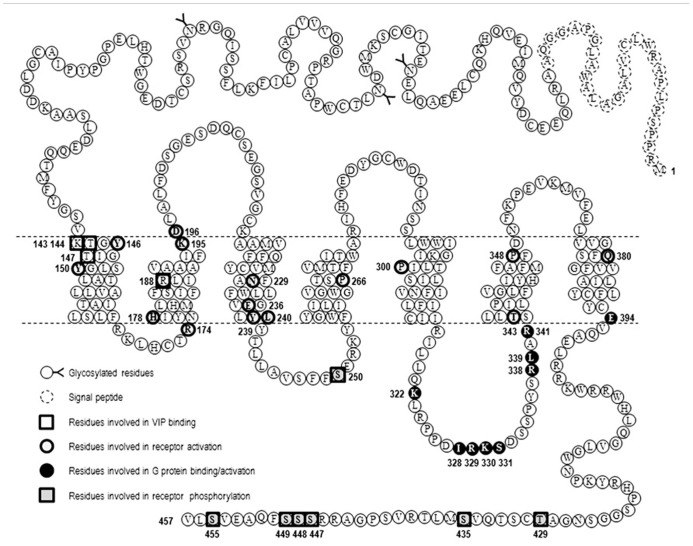
**Snake-plot representation of VPAC_1_ receptor**. Amino acid sequence of human VPAC_1_ receptor, the position of signal peptide, glycosylated residues, and amino acids important for VIP binding, receptor activation, and G protein coupling are also labeled.

As all members of GPCR-B family, VPAC receptors lack the E/DRY sequence. On the basis of subtle changes observed when Y^239^ and L^240^, located in TM3 of VPAC_1_, were substituted with alanine it was proposed that this YL sequence was equivalent to the E/DRY motif of GPCR-A family (Tams et al., 2001). Another model based on a three-dimensional analysis of the GLP-1 receptor proposed that a E/DRY motif could be formed by three non-adjacent residues consisting in R^174^ in the cytoplasmic end of TM2, E^236^, and Y^239^ in the distal part of TM3 of VPAC_1_ (Frimurer and Bywater, 1999). However, another study showed that Y^239^A, L^240^A, E^236^A, Y^239^A, and R^174^A mutants were undistinguishable from the wild type receptor (Nachtergael et al., 2006). One possible explanation for the discrepancy can be the fact that Tams et al. (2001) studied cyclic AMP measurements in intact cells a more sensitive model than the adenylate cyclase assay on membrane used in the other study. Nevertheless, even if the YL motif of GPCR-B family and E/DRY motif of GPCR-A family have the same location, they certainly do not have the same importance for receptor activation (**Figure 2**).

As mentioned before structural data confirmed that GPCR activation is accompanied by modification of helix conformation. For the β2-AR, the largest difference between the inactive and active structures is a large outward movement of TM6 and an inward movement of TM7 such as Tyr of the conserved NPxxY sequence (located in TM7) moves into the space occupied by TM6 in the inactive state. In GPCR-A family conserved prolines located in TM6 and laying near the ligand binding pocket and in TM7 (NPxxY sequence) play crucial role in these rearrangements due to their cyclic pyrrolidine ring side chain that introduce kink into the α-helical structure of TM and allow TM flexibility important for G protein coupling and signaling. In this line, a study of Knudsen et al. (2001) investigated the role of prolines located in TM of VPAC_1_ (**Figure 2**). They found that mutation of P266 (TM4), P300 (TM5), and P348 (TM6) into alanine significantly decrease VPAC_1_ expression levels but preserve VIP binding. P266A showed decreased ability to stimulate cAMP, while P300A and P348A displayed an increased potency in cAMP production combined with a high sensitivity toward GTP compared to the wild type receptor, thus demonstrating that these prolines are important for overall structure of VPAC_1_ and receptor activation (Knudsen et al., 2001).

**FIGURE 3:**
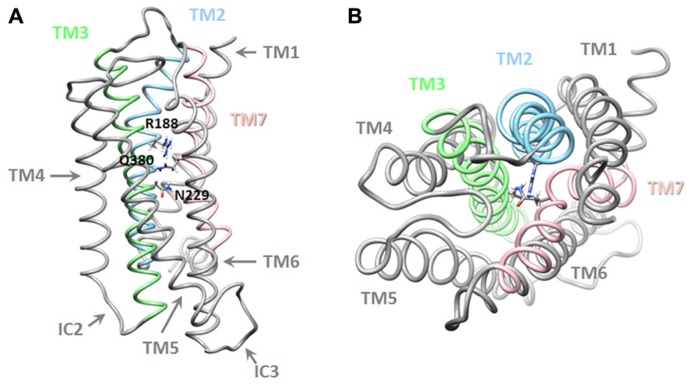
** Three-dimensional model of the transmembrane domains of the VPAC_1_ receptor**. Lateral **(A)**. and top **(B)**. view of a working model of human VPAC_1_ receptor, TM, and residues identified as important for receptor stabilization are also labeled. Details regarding modeling procedure are described in Chugunov et al. (2010).

A more recent study, that combined pharmacological and *in silico* approaches, identified a network of interactions between residues located in helices 2, 3, and 7 of the VPAC_1_ receptor, which could be involved in the stabilization of the receptor in absence of agonist and in early steps of receptor activation. It was proposed that, in absence of ligand, interaction between R^188^, N^229^, and Q^380^ ties helices 2, 3, and 7 together (**Figure 3**). Upon VIP binding, the interaction between R^188^ and N^380^ is broken and a stronger interaction (salt bridge) is established between R^188^ and the D^3^ side chain of VIP. TM2 and probably other helices undergo conformational changes causing key sequences located in intracellular loops to be exposed and to interact with the G proteins. In the meantime, the interaction network involving N^229^ and Q^380^ maintains TM7 in a conformation necessary for proper activation of G proteins. The three-dimensional model also suggested that Q^380^ could function as a floating “ferry-boat”, switching between R^188^ and N^229^ residues’ side-chains hence contributing to signal transduction propagation and activation of G proteins (Chugunov et al., 2010).

When considering other site-directed mutagenesis studies, it is likely that a complex and larger network of interaction between TM helices must be considered for stabilization of VPAC_1_ inactive and active conformations (**Figure 2**). Indeed, the mutation into arginine of H^178^ located at the bottom of TM2 led to a constitutively activated VPAC_1_ receptor (Gaudin et al., 1998). Similarly, mutation of T^343^, located at junction of the third intracellular loop and TM6 of VPAC_1_, into lysine, proline, or alanine also led to a constitutively activated receptor (Gaudin et al., 1999). Another study showed that Y^146^ and Y^150^, located in TM1 of VPAC_1_, do not interact directly with VIP but stabilize the correct active receptor conformation (Perret et al., 2002). It was also observed that K^195^ and D^196^ located at junction of TM2 and the first extracellular loop were essential for VPAC_1_ activation but were not directly involved in VIP recognition (Langer et al., 2003). How all these residues cooperate to propagate signal transduction after VIP binding remains to be elucidated and would require a model or a structure of the activated receptor in complex with VIP. Particularly the two N-terminal residues of VIP, H^1^ and S^2^, are likely to affect, directly or indirectly, the interaction network involved in receptor activation. In this line, a very recent study suggested that K^143^, T^144^, and T^147^, located in TM1 of VPAC_1_ could interact with H^1^ residue of VIP and play an important role in receptor activation (Ceraudo et al., 2012). Mutagenesis studies of VPAC_2_ are less exhaustive, however, some studies identified key residues involved in receptor activation such as Y^130^ and Y^134^ located in TM1 (Perret et al., 2002), K^179^ in TM2 (Vertongen et al., 2001), and N^216^ in TM3 (Nachtergael et al., 2006), suggesting that VPAC_1_ and VPAC_2_ share a similar pattern of activation. Moreover, so far as all residues that were identified as important for VPAC receptors activation are highly conserved among GPCR-B family members, they may therefore be involved in binding and activation mechanisms that are common to the whole family.

## MOLECULAR MECHANISMS INVOLVED IN VPAC/G PROTEIN BINDING AND ACTIVATION

The α subunit of heterotrimeric G proteins has a central role in interaction with both the receptor and the effectors. Several studies have shown that the C-terminal part of Gα subunit can directly bind to the receptor and is involved in the coupling specificity (Conklin et al., 1996). The current model of GPCR activation, based on the study of family A GPCRs, proposes that when the receptor switches to its active conformation, TM movements are accompanied by intracellular loops switches leading to exposure of the G protein binding pocket to cytosol and efficient binding to G protein. However, the diversity of sequences and loop sizes as well as their flexibility has made difficult the identification of a specific set of residues defining the coupling profile.

For the VPAC_1_ receptor, Gα binding domains are mainly located in the third intracellular loop (IC3) that contains subdomains dedicated to the recognition of the different Gα subunits. K^322^ located in proximal part of IC3 and E^394^ located at the junction of TM7 and the C-terminal tail are required for adenylate cyclase activation but not for the coupling to the inositol trisphosphate/calcium pathway. The former being involved in direct interaction with Gαs (G protein binding), as demonstrated by a reduced sensitivity to GTP, while E^394^ triggering switch of Gαs from inactive to active state (G protein activation; Couvineau et al., 2003; Langer and Robberecht, 2005). Similarly, two other sequences located in IC3 have been identified as important for VIP-induced intracellular calcium increase but not cAMP production. A small sequence, I^328^-R^329^-K^330^-S^331^, located in the central part of IC3 is involved in efficient binding of VPAC_1_ to Gαi/o and Gαq (Langer et al., 2002), while R^338^ and L^339^, located at the distal part of IC3, mediate interaction of VPAC_1_ with Gαi/o (Langer and Robberecht, 2005). Combining mutations in the proximal and distal part of IC3 together with mutation of E^394^ gave rise to a completely inactive VPAC_1_ receptor with respect adenylate cyclase activation and intracellular calcium increase (**Figure 2**). In VPAC_2_ receptors, L^310^ located in the proximal part of IC3 contributes to Gαs activation and the proximal part of IC3 (R^325^ and K^328^) is involved in both Gαs and Gα16 coupling. The combined mutations of these three amino acids generates an inactive VPAC_2_ receptor with respect to [Ca^2^^+^]_ i_ increase and adenylate cyclase activation (Langer et al., 2005).

Among the different members of the GPCR-B family, proximal and distal domains of IC3 share conserved sequences that could therefore represent common G protein binding motifs. In line with this hypothesis, studies performed on other members of the GPCR-B family identified the proximal domain of IC3 as essential for adenylate cyclase activation but the amino acids involved may differ and additional conserved sequences located in other intracellular regions of the receptor may also be necessary as seen for glucagon (IC2; Cypess et al., 1999) and CGRP receptors (R^151^ located in IC1; Conner et al., 2006). The junctions of IC3 loop are predicted to be α-helical and it is assumed that the correct positioning of charged amino acids plays an important role in G protein interaction. However, other data suggest that lipophilic and aromatic residues are also important for G protein interaction. It is possible that IC3 loop junctions activate G protein directly or that they may serve as regions that control the loop conformation. As mutations may change both direct interaction site and secondary structure, it is difficult to define more precisely the mechanisms involved in IC3 loop/G protein interaction.

## REGULATION OF VPAC RECEPTORS ACTIVITY

Besides coupling to the effectors, GPCR activation by agonists also initiates the process of receptor desensitization, an adaptive response contributing to rapidly fade the G protein signaling. This process starts with phosphorylation of the receptors in intracellular loops and carboxyl terminus by activity dependent kinases (PKA and PKC) and/or by receptor activated dependent kinases (GPCR kinases or GRK). GRK mediated phosphorylation of the receptor promotes the high affinity binding of β-arrestins to the receptor, which both sterically interdicts further coupling of G protein and may act as a signal transducer to activate for instance MAPKs, AKT, and PI3 kinases. β-arrestins are also able to bind proteins of the endocytic machinery including clathrin and adaptor protein AP2 and promotes receptor internalization (Shenoy and Lefkowitz, 2011).

**FIGURE 4:**
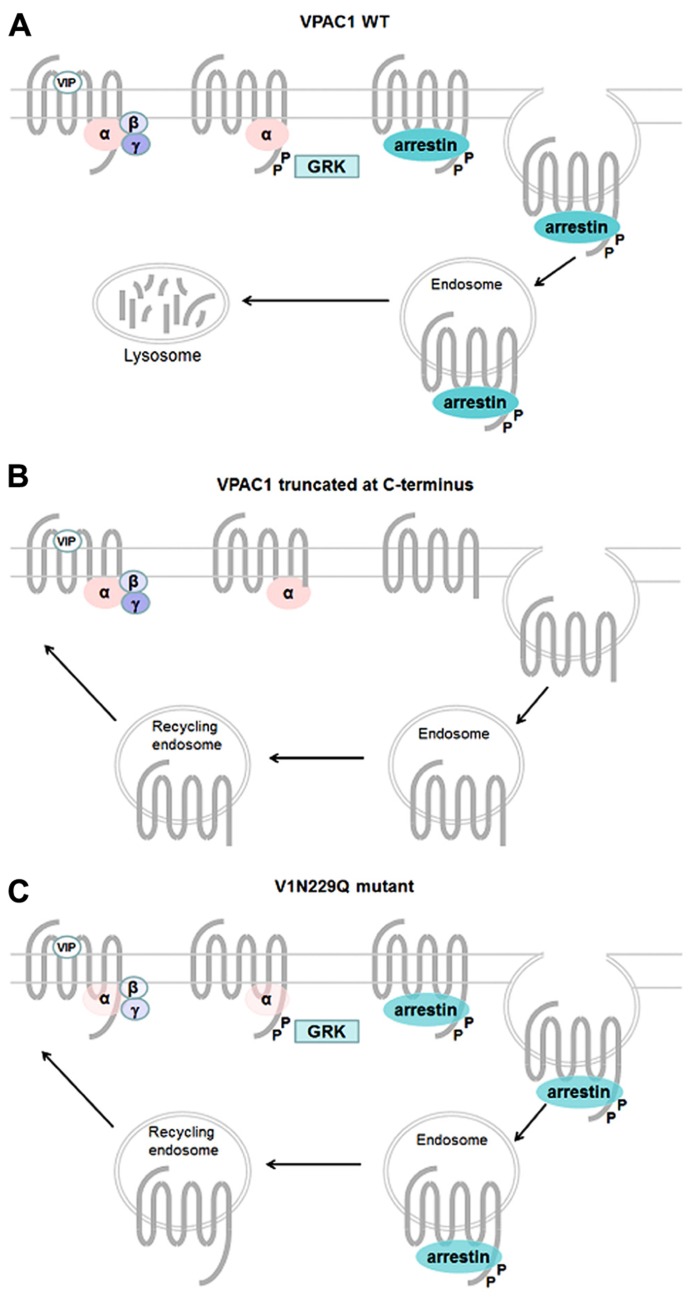
**Mechanisms involved in regulation of VPAC_1_ activity**. Upon VIP binding wt VPAC_1_ **(A)**. is rapidly phosphorylated through a GRK-dependent mechanism and desensitized, phosphorylation promotes high affinity binding of β-arrestin to VPAC_1_ and internalization into endosome through an arrestin- and dynamin-dependent mechanism. After internalization VPAC_1_ is not re-expressed at the cell surface. In some truncated VPAC_1_ receptors **(B)**., VIP fails to induce GRK-mediated receptor phosphorylation but the receptor is still able to internalize through an arrestin-independent mechanism and is re-expressed at the cell surface. Some VPAC_1_ receptor mutants **(C)**., with decrease in potency and efficacy of VIP-stimulated adenylate cyclase activity, preserved receptor recognition of agonists and antagonist and a preserved sensitivity to GTP, are still phosphorylated and internalized and recycle back to cell surface.

Like most GPCRs, VPAC_1_ and VPAC_2_ receptors are rapidly phosphorylated after agonist exposure. Absence of inhibitory effect of PKA and PKC inhibitors (Langer et al., 2005; Langlet et al., 2005), use of dominant negative GRK and GRK over-expression experiments (Shetzline et al., 2002; Huang et al., 2007), suggest that GRK are the main kinases involved in VIP receptors phosphorylation. VPAC_1_ receptor phosphorylation induces β-arrestin translocation to cell membrane, however, over-expression of β-arrestin in HEK 293 cells only causes a minor decrease in cAMP, suggesting VPAC_1_ desensitization may occur through an arrestin-independent mechanism (Shetzline et al., 2002). By using site-directed mutagenesis studies, T^429^, S^435^, S^447^, S^448^, S^449^, S^455^ located in the C-terminus and S^250^ located in IC2 were identified as potential candidates for VIP-induced VPAC_1_ receptor phosphorylation (Marie et al., 2003; Langlet et al., 2005; **Figure 2**). Combining the mutations of these identified residues indicated that the effect on phosphorylation was not additive and the mutants tested maintained a phosphorylation level of about 30% of that observed for the wild type receptor except when all the Ser/Thr residues of the C-terminus were mutated (Langlet et al., 2005). Although it was shown that VPAC_1_ forms a complex with β-arrestin and this complex is transported into the cell during endocytosis (Shetzline et al., 2002), direct correlation between the VPAC_1_ phosphorylation level and internalization was not obvious as in some mutated receptors a significant reduction in phosphorylation has no or little effect on receptor internalization. For instance, some VPAC_1_ truncated receptors were still internalized while VIP-induced phosphorylation was undetectable thus suggesting an arrestin-independent mechanism of internalization and only mutation of all the Ser/Thr residues of the C-terminus and S250 completely abolished VPAC_1_phosphorylation and internalization (Langlet et al., 2005). VPAC_1_ and VPAC_2_ receptors are both rapidly internalized following agonist exposure but differ in their trafficking pattern. After internalization, VPAC_1_ receptors are not re-expressed at the cell surface within 2 h after agonist washing (suggesting that VPAC_1_ binds with high affinity β-arrestin) while VPAC_2_ receptors are recycled back to the cell surface (suggesting that VPAC_2_ binds β-arrestin with low affinity; Langlet et al., 2004, 2005). In VPAC_2_ receptors, it was found that inactivating mutations that alter G protein coupling reduced both receptor phosphorylation and internalization in a manner that appeared directly linked to the alteration of the Gαs and Gα16 coupling (Langer et al., 2005). As mutants studied do not affect phosphorylatable residues, this suggests that impaired receptor phosphorylation and internalization directly reflect the reduced ability of the mutants to adopt active receptor conformation. However, another study showed that VIP stimulated phosphorylation of N^229^Q VPAC_1_ and N^216^Q VPAC_2_ (two mutants characterized by a decrease in potency and efficacy of VIP stimulated adenylate cyclase activity, by the absence of agonist stimulated [Ca^2^^+^]_ i_ increase, by a preserved receptor recognition of agonists and antagonist and by a preserved sensitivity to GTP) was only slightly decreased, that receptor internalization was comparable to that of the wild type receptors but unlike wild type VPAC_1_ the N^229^Q mutant was rapidly re-expressed at the cell surface upon VIP washing (Nachtergael et al., 2006). As N^229^and N^216^ residues are located in the middle of TM3, it seems unlikely that they could be in direct interaction with β-arrestin but probably contribute to receptor conformation necessary for high affinity binding with β-arrestin. This later study thus suggests that receptor conformation necessary for activation and regulatory mechanisms could be different (**Figure 4**). As mentioned before, β-arrestins are also able to act as a signal transducer and activate G protein-independent signaling pathways but to date such a mechanism has not been described for the VIP receptors.

## VPAC RECEPTORS OLIGOMERIZATION

It is now well accepted that, like for single transmembrane receptors, GPCRs are also able to form oligomeric complexes either with themselves (homo-oligomerization) or with other receptors (hetero-oligomerization) that may affect the properties of the receptor. When considering the large repertoire of GPCRs, and thus the multitude of potential oligomers that can be formed, a challenging task consists in identifying the specificity and the physiological relevance of the partners, and the functional consequences of these associations. By far, the GPCR-A/rhodopsin family has been the most extensively studied, and data collected indicate that the consequences of oligomerization may be detected at the level of both physiological and pharmacological ligand recognition, in signaling properties as well as at desensitization and internalization levels. Besides pharmacological and functional regulation, it was also demonstrated that GPCR oligomerization plays a role in receptor maturation and in expression at the cell surface (Milligan, 2009; Maurice et al., 2011).

Regarding VPAC receptors, two studies performed on the prostate cancer cell line LNCaP (Juarranz et al., 2001) and adipocytes (Akesson et al., 2005) demonstrated that those cells expressed both VPAC_1_ and VPAC_2_ receptors but differed in their pharmacological response with respect to the selective VPAC_1_ and VPAC_2_ agonists. LNCaP cells displayed a VPAC_1_-like phenotype (the VPAC_2_ selective agonist being inactive) while adipocytes displayed a VPAC_2_-like phenotype (the VPAC_1_ selective agonist being inactive). One possible explanation of these results might be the formation of inactive receptors dimers neutralizing the receptor expressed at the lowest concentration and letting only one receptor active. First evidence that VPAC receptors form oligomers came from a study of Harikumar et al. (2006) who demonstrated by using biophysical methods that VPAC_1_ and VPAC_2_ receptors formed constitutive homo- and hetero-oligomers but also heterodimers with secretin receptors that remained trapped into the cells (Harikumar et al., 2006). In an attempt to evaluate the functional consequences of hetero-oligomerization of VPAC receptors, another study investigated the effect of coexpressing VPAC_1_ and VPAC_2_ receptors in Chinese hamster ovary (CHO) cells on ligand binding, adenylate cyclase activation, receptor internalization, and trafficking. Three agonists were used, the natural ligand VIP, and selective VPAC_1_[K^15^,R^16^,L^27^]VIP(1-7)/GRF(8-27)] and VPAC_2_[Ro 25-1553] agonists. They found that pharmacological properties of cells expressing both receptors were not different from those obtained when mixing cells expressing each receptor individually. Similarly, VIP receptors co-expression did not modify receptor internalization and trafficking patterns following exposure to VIP or selective agonists (Langer et al., 2006). Although this study did not point out any pharmacological consequences of constitutive heterodimerization of VPAC_1_–VPAC_2_ receptors, it suggests that the pharmacological profile of the selective VPAC_1_ and VPAC_2_ receptors ligands that was established in cells expressing one receptor subtype is also applicable to cells that could express both receptors endogenously. Thus making possible the activation, antagonism, or downregulation of one receptor subtype without affecting the other. Additional experiments are now needed to demonstrate that these oligomers do exist in more physiological conditions and identify physiological consequences, if any, of VIP receptors oligomerization.

Some GPCRs are also able to form hetero-oligomers with receptor activity-modifying proteins (RAMPs), a family of single transmembrane accessory proteins (RAMP1, 2, and 3). More particularly, hetero-oligomerization of RAMPs with two GPCR-B family members, the calcitonin receptor and the calcitonin receptor-like receptor, generates multiple receptor phenotypes with different specificities for endogenous ligands. It has first been shown that VPAC_1_ receptors, but not VPAC_2_ receptors, were also able to interact with RAMP1, 2, and 3. VPAC_1_–RAMP complexes did not display altered ligand specificity compared with VPAC_1_ alone. However, specific interaction with RAMP2 led to a significant enhancement of VIP- and PACAP-induced inositol phosphate hydrolysis with unaltered cAMP production (Christopoulos et al., 2003). A more recent study, however, observed that VPAC_2_increases cell surface expression of all three RAMPs. As seen for VPAC_1_, VPAC_2_, and RAMP co-expression had no or little effect on agonist-stimulated cAMP production but RAMP1 and RAMP2 significantly enhanced basal coupling to Gαi (Wootten et al., 2012). The RAMPs being largely distributed throughout the nervous system and peripheral tissues, it would be interesting to investigate more deeply the properties of VPAC–RAMP complexes such as for example selective agonists and antagonists specificity, receptor signaling through other pathways, receptor desensitization and internalization.

## CONCLUSION AND PERSPECTIVES

Vasoactive intestinal peptide plays diverse and important role in the CNS, PNS, gastrointestinal tract, and immune functions and VPAC receptors constitute potential targets for the treatment of several diseases such as neurodegenerative disorder, schizophrenia, asthma, diabetes, gastrointestinal motility disorder, Crohn’s disease, and rheumatoid arthritis. A key limiting factor in this field is that all the currently useful pharmacological tools are peptides, and thus limit their use in human therapy. The first small molecule antagonists of VPAC_1_ (Harikrishnan et al., 2012) and VPAC_2_ (Chu et al., 2010) have been described recently and emerge from high throughput screening of compound collection. However, all are low affinity, micro molar range, antagonists and need further improvement to expect to obtain druggable modulators of VPAC receptors activity. Better understand of structure, ligand binding site, and molecular pharmacology of VPAC receptors constitute thus a key information for the rationale design of VPAC receptors modulators and potential druggable compounds.

## Conflict of Interest Statement

The author declares that the research was conducted in the absence of any commercial or financial relationships that could be construed as a potential conflict of interest.
